# Multiparametric Monitoring in Equatorian Tomato Greenhouses (III): Environmental Measurement Dynamics

**DOI:** 10.3390/s18082557

**Published:** 2018-08-04

**Authors:** Mayra Erazo-Rodas, Mary Sandoval-Moreno, Sergio Muñoz-Romero, Mónica Huerta, David Rivas-Lalaleo, José Luis Rojo-Álvarez

**Affiliations:** 1 Departamento de Eléctrica y Electrónica, Universidad de las Fuerzas Armadas ESPE, Av. General Rumiñahui s/n, Sangolquí 171-5-231B, Ecuador; drrivas@espe.edu.ec; 2 Departamento de Teoría de la Señal y Comunicaciones, Sistemas Telemáticos y Computación, Universidad Rey Juan Carlos, 28943 Fuenlabrada, Spain; sergio.munoz@urjc.es (S.M.-R.); joseluis.rojo@urjc.es (J.L.R.-Á.); 3 Departamento de Ciencias Exactas, Universidad de las Fuerzas Armadas ESPE, Av. General Rumiñahui s/n, Sangolquí 171-5-231B, Ecuador; mjsandoval@espe.edu.ec; 4 Center for Computational Simulation, Universidad Politécnica de Madrid, Boadilla del Monte, 28660 Madrid, Spain; 5 Carrera de Telecomunicaciones, Universidad Politécnica Salesiana, Cuenca 010105, Ecuador; mhuerta@ups.edu.ec

**Keywords:** greenhouses, rhythmometric, parametric modeling, residuals, mutual information

## Abstract

World population growth currently brings unequal access to food, whereas crop yields are not increasing at a similar rate, so that future food demand could be unmet. Many recent research works address the use of optimization techniques and technological resources on precision agriculture, especially in large demand crops, including climatic variables monitoring using wireless sensor networks (WSNs). However, few studies have focused on analyzing the dynamics of the environmental measurement properties in greenhouses. In the two companion papers, we describe the design and implementation of three WSNs with different technologies and topologies further scrutinizing their comparative performance, and a detailed analysis of their energy consumption dynamics is also presented, both considering tomato greenhouses in the Andean region of Ecuador. The three WSNs use ZigBee with star topology, ZigBee with mesh topology (referred to here as DigiMesh), and WiFi with access point topology. The present study provides a systematic and detailed analysis of the environmental measurement dynamics from multiparametric monitoring in Ecuadorian tomato greenhouses. A set of monitored variables (including CO${}_{2}$, air temperature, and wind direction, among others) are first analyzed in terms of their intrinsic variability and their short-term (circadian) rhythmometric behavior. Then, their cross-information is scrutinized in terms of scatter representations and mutual information analysis. Based on Bland–Altman diagrams, good quality rhythmometric models were obtained at high-rate sampling signals during four days when using moderate regularization and preprocessing filtering with 100-coefficient order. Accordingly, and especially for the adjustment of fast transition variables, it is appropriate to use high sampling rates and then to filter the signal to discriminate against false peaks and noise. In addition, for variables with similar behavior, a longer period of data acquisition is required for the adequate processing, which makes more precise the long-term modeling of the environmental signals.

## 1. Introduction

In 2017, the population on Earth was 7.7 billion [1]; whereas, in the last decade, the growth rate of the population has slowed, the trend is to continuously increase, and the least developed countries still exhibit rapid growth. It is expected that, by 2050, the world population will reach 9 billion people [2,3]. Currently, the growth of the world population brings unequal access to food as an effect; crop yields are not increasing as fast as population, and a result is that food demand will not be satisfied in the future [4,5]. The challenge is to be able to focus on the use of food crops and to minimize crops oriented to bio-fuels, hence increasing crop yields. For this reason, many research centers are focused nowadays on applying optimization and analysis techniques to the agricultural sector [6,7], specifically in crops with greater demand, such as potatoes, corn, rice, or tomatoes, among others [8,9,10,11].

In this study, we focus on tomato crops because it is one of the most consumed and appreciated vegetables in the world, due to its high content in carotene, a natural antioxidant. In 2017, the world production was approximately 177 million tons [1], which emphasizes the relevance of optimizing tomato crops by applying precision agriculture. One of the key technological aspects in this setting is the current possibility of monitoring climatic variables with sensor networks, which is receiving increasing attention. In the first companion paper [12], we described the analysis of the WSN topologies and their configuration, in terms of the design and implementation of hardware and software for the nodes with different communication technologies. For this purposes, three WSNs were designed and implemented: ZigBee technology with star topology, ZigBee with mesh topology (referred to here as DigiMesh); and WiFi technology (access point topology). In the second companion paper [13], we presented a detailed analysis of the dynamics of the energy consumption in those three WSNs for tomato greenhouse monitoring in Ecuador. To this aim, the statistical patterns of energy consumption were studied with detail in DigiMesh, WiFi, and ZigBee WSNs.

Surprisingly, and to our best knowledge, few studies have focused on the analysis of all the data that are currently obtained from the environment variables in greenhouse scenarios and applications. Some of them have applied predictive techniques for energy saving in greenhouses, using neural networks such as the multilayer perceptron [14], or they have aimed to reduce the large number of duplicated and redundant data transmission [15]. Systems such as the Modified Extended Linearized Predictive Controller have been proposed, which use non-linear modeling techniques to control the greenhouse air temperature of usual parameters, namely, heating and natural ventilation [16]. Other approaches have developed energy-saving techniques in WSN by analyzing the energy consumption changes with the frequency of the transmitted measurements by the sensors and to characterize the send/receive configuration of the radio-frequency modules. Effort has also been devoted to choosing communication protocols with lower communication rates [17] and to controlling facilities in greenhouses by remotely using short message services [18], which could also influence positively the sensor battery lifetime. Nevertheless, little work is available on the dynamics of a variety of environmental variables in greenhouses, on the convenient sampling rate to adequately scrutinize these dynamics, and on the information that is either specific or shared in some sense for each environmental variable compared with others.

Therefore, the present study aimed to provide a detailed and basic analysis of the environmental measurement dynamics in multiparametric monitoring performed at Ecuadorian tomato greenhouses. We used suitable statistical analysis tools to better scrutinize and study the dynamics of a variety of simultaneously recorded environmental variables. From a time-process analysis viewpoint, we used tools for scrutinizing the statistical nature of the recorded signals in terms of their cyclo-stationarity, namely, rhythmometric analysis and residual analysis (in terms of scatterplots, Bland–Altman plots, and time evolution of the residuals) [19]. In addition, the cross-information among simultaneously measured variables is analyzed, both in terms of simple representations, such as scatterplots and Bland–Altman plots, as well as using mutual information descriptions. For this purpose, an experimental setup was established considering a variety of monitored variables, all of them recorded at high-rate sampling conditions.

The scheme of the rest of the paper is as follows. Section 2 presents existing research works on the environmental measurement dynamics of tomato greenhouse monitoring based on WSNs. Section 3 presents a short summary of the system proposed in the companion papers. Section 4 presents the details on the statistical tools used to analyze these variables. Section 5 shows the detailed results of the analysis, starting from a description of the practical methodology, and then focusing on the rhythmometric model adjustment considerations to every variable. The cross-variable information is also scrutinized in terms of the spacial diversity, network diversity, and other relevant factors. Finally, in Section 6, discussion is presented and conclusions are established.

## 2. Related Work

Data analysis tools have been widely applied in diverse fields, such as health [20], industry [21], or agriculture [22]. In agriculture, working with monitoring data allows optimizing water usage using genetic algorithms [23], analyzing crops status using images [24,25], detecting weather related risks [26], studying greenhouse indoor air quality [27], or minimizing plague impact in crops [28], among many others.

There is not so much research on the dynamics of environmental variables in greenhouses. Some existing studies use neural networks, genetic algorithms, and artificial intelligence techniques [29,30]. Predictive analytics help producers to make decision for pests or lack of nutrients in crops [31,32], to reduce energy consumption by analyzing communication protocols [33], or to better choose the data type to be transmitted. Other studies have focused on dynamic crop models, specifically evaluation, analysis, parameterization, and applications [34]. In [35], the impact of the number of crop state variables and their measurement errors on the prediction skills is analyzed with statistical models.

Table 1 shows a compilation of research work on the dynamics of environmental variables in greenhouses. The analyzed articles focus on evaluating several relevant environmental variables in greenhouses, such as air temperature, air relative humidity, soil temperature, soil moisture, illumination, conductivity, water-level, pH value, or CO${}_{2}$. In summary, the proposed solutions for the analysis of the dynamics of environmental variables in greenhouses often apply neural networks, genetic algorithms, predictive models, statistical analysis, estimation methods, Bayesian networks, machine learning, and data mining techniques. Researchers indicate that traditional experimental and statistical methods do not have good results when applied on agricultural big data, while machine learning exhibit interesting options for the analysis of big data [36]. However, whereas this conclusion in the literature is interesting, there is a lack of basic analysis of the environmental variable dynamics with time-process analysis tools, providing basic information about the variables and their cross-relationship. To our best understanding, this kind of analysis should be addressed with detail before moving towards more advanced prediction techniques to have a solid description of the time-processes nature at hand. Our work is an opportunity to analyze the trends and time-series dynamics in agricultural data acquisition and processing from the unreported statistical description in the literature.

## 3. Summary of the Proposed System for Tomato Greenhouse Monitoring

**Figure 1:**
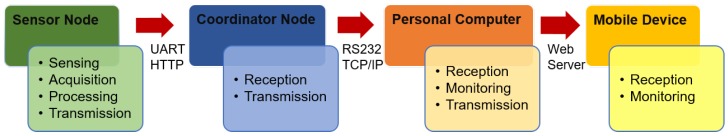
General description of the elements, processes, and communication protocols for the WSN systems in this work.

In the first of the companion papers [12], a system is proposed to characterize the performance of the ZigBee (star and mesh topology), and WiFi standards for monitoring greenhouses. We summarize here the fundamental elements to understand the present work. Figure 1 shows a general scheme of the system for the three WSNs, which consists of a set of sensor nodes, a Coordinator Node, a personal computer, and a mobile device. The sensor nodes installed inside the tomato greenhouse allow the data acquisition from the sensors of several variables, such as air relative humidity, luminosity, air temperature, solar radiation, ultraviolet radiation (UV), wind speed, wind direction, and CO${}_{2}$.

The acquired data were conditioned and used for the creation of the data packages or frames, and then, these frames were transferred from the Waspmote card to the communication module, where they were wirelessly transmitted to the Coordinator Node. This last one transmitted the environmental data of each sensor to the personal computer containing the Human–Machine Interface (HMI) designed in LabVIEW${}^{\mathrm{TM}}$, and the mobile device allowed the user to visualize the data for each variable and for each WSN node.

Sensors were selected according to the environmental variables that most affect the tomato growth. They were low consumption and compatible with processing cards. The data acquisition used the PRO 2.0 agriculture card (air temperature, solar radiation, luminosity, air relative humidity, wind speed, wind direction, and UV radiation) and the gas card PRO 2.0 (CO${}_{2}$). These cards have very low power consumption. The Waspmote card with low energy consumption processed the data. The internal memory of the Waspmote stored the data after being processed, and data packets were transferred to the communication module by UART serial controller (for DigiMesh and ZigBee) or by HTTP protocol (for WiFi).

The reception and transmission of the Coordinator Node packets used the communication modules XBEE ZB S2 PRO and XBEE ZB S1 PRO (for ZigBee and DigiMesh, respectively), with low energy consumption. The transmission speeds (9600, 19200 and 57600 bauds) were configured according to the operation rates of the Waspmote modules. The tree topology of the ZigBee network has the disadvantage that, if one of the nodes loses communication with the Coordinator Node, then it will be permanently out of service. The mesh topology of the DigiMesh network was implemented using redundancy, where each sensor node can communicate directly with the Coordinator Node or through another sensor node. The WiFi network receives and transmits the packets through the RN-XV wireless communication module, which has low power consumption. The ZigBee and DigiMesh networks transmit the data packets to the PC using the RS-232 interface, and the WiFi network uses the TCP/IP protocol.

The packages received in the personal computer were routed to LabVIEW${}^{\mathrm{TM}}$. The database was read and stored in LabVIEW${}^{\mathrm{TM}}$ through toolkits for creating, opening, and closing the communication channel, as well as for calling and storing data. The monitoring of the variables was made with the graphic interface developed in LabView according to the design stages, such as data separation, error detection, alarm generation, or variable monitoring. The interface was similar for the three networks and the user was able to know the real-time and precise values of each variable of the tomato greenhouses. The transmission of the data hosted in the MySQL acquisition station was controlled by the interaction with the web server.

The Apache web server connected to the MySQL database to access the smartphone mobile application by means of a webpage. This application was developed with the Eclipse Integrated Development Environment (IDE) and it used the Android development environment software development kit (SDK). The user had access to the interfaces for the data generated in the greenhouse by three WSNs, from any geographical location with the availability of the mobile device.

**Figure 2:**
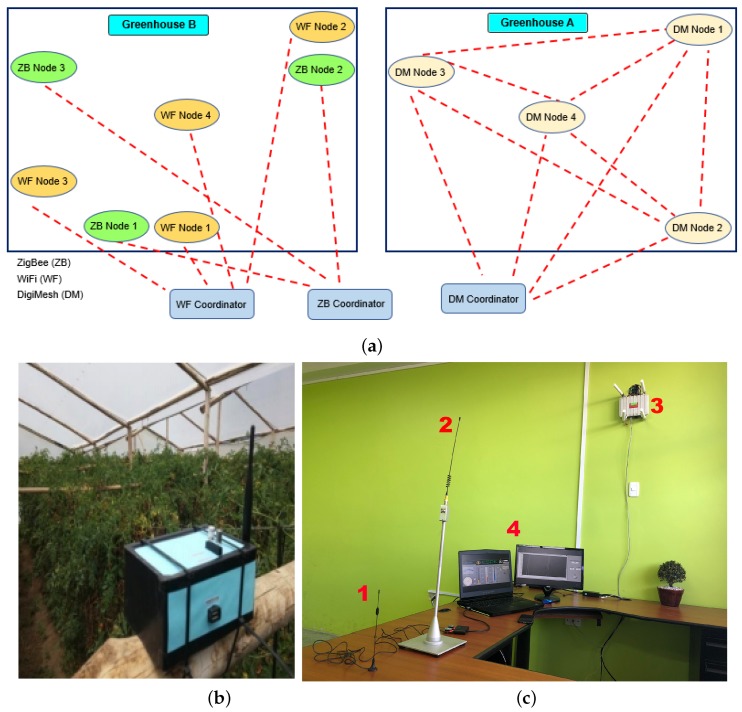
Description of the experimental set-up: (**a**) map of the location of the sensor nodes in the two tomato greenhouses; (**b**) sensor node in the greenhouse; and (**c**) coordinator nodes and data storage station.

The experimental environment consists of two tomato greenhouses, which are located in the area of Salcedo, at coordinates −1.018373, −78.583888. The first one (Greenhouse A) had an area of 4000 m${}^{2}$ (80 m length, 50 m width), was sawtooth type, with plants in flowering stage, and was installed with the DigiMesh network, while the second one (Greenhouse B) had an area of 3500 m${}^{2}$ (70 m length, 50 m width), was curve type, with plants in harvest stage, and was installed with the ZigBee and WiFi networks. Figure 2a shows the distribution of the nodes in both greenhouses, Nodes 1–3 were installed at the contour of the greenhouses, and Node 4 was installed in the center. Figure 2b shows a Sensor Node; Figure 2c shows the laboratory configuration: the ZigBee Coordinator Node (1); the DigiMesh Coordinator Node (2); the WiFi router (3); and the monitoring and control station (4).

## 4. Statistical Analysis of Environmental Measurement Dynamics

In this section, we describe the data analysis tools that we used to study the environmental variable dynamics. From a time-process analysis viewpoint, we use dtools for parametric modeling of every variable in terms of their cyclo-seaonality (rhythmometry analysis and bootstrap method for its order selection) and residual analysis for these models (Bland–Altman graphs, temporal evolution of the residuals, and residual histograms). We also scrutinized the dependence between measurement pairs (either the same variable in different sensors or networks, or different variables), yielding a simple statistical description in terms of scatterplots and Bland–Altman plots, and an advanced analysis in terms of their mutual information (MI). The mathematical notation described in [13] for the bootstrap estimation method and histograms can also be used in this study, and, in this paper, we extend it and describe the theoretical framework for the statistical analysis tools set specific for this work.

Rhythmometric analysis is a statistical framework that can be applied to the automatic extraction of circadian, infradian, and ultradian seasonal components of a time series, based on a hypothesis test generated by the bootstrap resampling technique [49]. Ultradian rhythms are repetitive cycles that occur during and within a day, while infradians correspond to events whose period is longer than 24 h, which can be interpreted as they occur less than once a day [50]. In this study, we used this tool to adjust each environmental variables signal acquired during four days to a rhythmometric model. For this purpose, we applied the Cosinor temporal regression model, which is defined as a statistical technique for estimating cosenoidal models adjusted to different types of temporal data whose sampling may be unequally distributed, and which is widely used in the representation of the chronobiological oscillation of a variable with rhythmic behavior [51]. For the analysis of environmental variables, we used this analysis tool, since its behavior is very similar to those described in the revised scientific literature. Note that, after finishing the model adjustment process, the residual time series can be estimated, which are extremely useful for model diagnosis. For instance, it is possible to scrutinize their time distribution, so that time-biased structures can be observed, or their statistical distribution, by means of the histograms to provide us with a view of the model suitability. The detailed equations of the statistical method followed here are compiled in Appendix A.

The scatterplot is a type of statistical graph designed to illustrate the relationship between two data signals. The construction of a scatterplot consists of the graphical representation of a reference signal (*X*-axis), and a dependent signal (*Y*-axis) [52]. In the case of sample observations given by pairs of simultaneously observed time series, $\left(y_{tr},{\hat{y}}_{tr}\right)$ (r=1,2,3,…,R), these pairs are plotted in the rectangular coordinate system to obtain the scatterplot, which helps the investigator to visualize the form of mathematical relationships, trends or structures that follow the signals jointly. For example, if the points approximate a straight line, then there is a linear relationship between the two signals [53].

An additional comparison tool is the Bland–Altman plot, which is a graphical technique for comparing two different sets of signals. This plot can be used in two ways. On the one hand, it allows comparing two measurement instruments, given in this case by pairs $y_{n}^{1}\left(tr\right),y_{n}^{2}\left(tr\right)$ referring to a same magnitude as measured by two different sensors. If none of those sensors can be considered as a gold standard, then the *X*-axis is given by their average, $0.5(y_{n}^{1}\left(tr\right)+y_{n}^{2}\left(tr\right))$, and the *Y*-axis is given by their difference, $\left(y_{n}^{1}\left(tr\right)-y_{n}^{2}\left(tr\right)\right)$. The resulting transformed signals are represented as their scatterplot. In addition, Bland–Altman plot is a visual check that the magnitudes of the differences are constant over the entire measurement range [54,55].

On the other hand, Bland–Altman plots can also be used as a tool for model diagnosis, which are complementary to residual distribution analysis and temporal analysis. In this case, the *X*-axis represents the actually measured magnitude $y_{n}$, while the *Y*-axis represents the associated residuals $e_{n}$. The presence of model nonlinearity, model bias, heteroscedasticity, and others, can often be readily identified in these cases. Appendix A includes an extended notation for scatterplots and Bland–Altman plots.

The MI is a measurement of the dependence between two random variables, in the sense that it specifies the amount of information that can be obtained from one random variable from knowing the other [56]. This concept is closely related to the entropy concept defined in Information Theory for a single random variable, which defines the amount of information that is explained by that variable. Whereas the correlation coefficient is limited to linear relationships between two real-valued variables, the MI gives a more general description in terms of the measurement of the similarity between the joint distribution of two general random variables and the product of their factored marginal distributions. The reader can see Appendix A for a more detailed explanation and notation on the MI methods used in this work.

MI has been used in a number of practical applications, for instance, to evaluate how the environmental variables independently influence the ecosystem service interaction [57], or to investigate the relationship between land surface temperatures and the spatial pattern of green space [58]. In our scenario, we propose to perform a similar interaction analysis between the environmental variables of a tomato greenhouse using MI in different situations and conditions, for instance, sensors in similar locations but different networks, different sensor locations in the same greenhouse, or different variables. Note that the scatterplot provides us with a simple statistical description of the relationship among pairs of variables, which is comparable to an empirical estimation of the joint distribution of two environmental variables, whereas the MI can provide us with an information-theory based quantification of the possibly non-linear relationships between them, while retaining a similar theoretical basis for their comparison.

## 5. Experiments and Results

### 5.1. Methodology Description

The statistical methods described in the previous section were applied to study the acquired environmental measures given by air temperature, air relative humidity, CO${}_{2}$, luminosity, wind speed, solar radiation, UV radiation, and wind direction. All of these variables were monitored in a tomato greenhouse during four days using three WSNs, two with ZigBee technology (star and mesh topology), and one with WiFi technology (access point topology). The obtained monitored signals were modeled by using the described LS rhythmometric method with fundamental period $T_{o}$ = 24 h. For every model we included some ultradian spectral components, and only one infradian component (approximate occurrence period of 40 h), because these frequencies are relevant in studies where the observed samples correspond to occurrence periods of several days or weeks. Moreover, fluctuations were considered as possible in each ultradian frequency component. The significant components of the model were automatically selected, according to the described procedure of their inclusion in decreasing amplitude order until the bootstrap test identified them as non-significant.

Ad-hoc software was developed in Matlab${}^{\mathrm{TM}}$, and the analysis tool included two parameters that need to be set by the user to improve the accuracy of the models. The first parameter to scrutinize is the regulation factor (Reg) that compensates for the presence of ill-conditioning of the inverted matrix used in the LS adjustment model. This Reg parameter was considered appropriate when the signal provided by the rhythmometric analysis accurately followed the measured samples, making sure that it was not an overfitted model also learning the noise, and if the general trend of the estimation compared to the original signal was not lost. Accordingly, the model adjustment was considered acceptable when the resulting estimated signal was generally situated in the midway of measured samples, and hence the residuals were symmetrically distributed around zero (solutions with reduced bias). The second parameter to scrutinize is the order of a mean filter (Ord), which was used to attenuate the noise in those variables whose amplitude rapidly changed, as high volatility was especially observed when working at reduced sampling rates. This was especially noticeable in variables such as wind speed, luminosity, and CO${}_{2}$. Parameter Ord was set on the basis that the filtering did not distort the visually significant trends in the signal, and that the modeled signal did not deviate from the visually observed sample patterns.

For the model validation, we used the Bland–Altman plots, the residual histograms, and the residual temporal evolution. By using the Bland–Altman graphs, it was possible to determine the noise distribution or precision between the acquired samples in each variable and the estimated model. For this purpose, concordance limits were established as usual in this methodology, and the model was considered as adequate if the majority of the compared samples fell inside them, they were near to zero, and they showed no heteroscedasticity pattern. The model statistical mismatch was also scrutinized by means of the histograms, accounting for the relative frequency distribution of the systematic error and considering the model as successful when the residual distribution trended to be Gaussian (or at least unimodal), and its standard deviation was narrow enough compared to the order of magnitude of the environmental signal fluctuation, whereas the model was considered out of adjustment otherwise. The residual time distribution complemented the validation by identifying temporal regions in those cases where bias could be observed for some time periods in the signal and in the adjusted model.

The joint distribution between pairs of variables was scrutinized in terms of their scatter diagrams. Specifically, we compared two signals of a particular environmental variable, acquired by nodes of the same or different network. To complement the comparative analysis of variables, we finally included MI calculations among all the variables to identify the dependence between pairs of environmental signals, according to the nodes spatial location in the greenhouses, the communication technology type of each network, and the filtering incidence.

### 5.2. Rhythmometric Model Adjustment

The rhythmometric model was adjusted for the environmental variables in terms of the model order and the regularization parameter. The interested reader can find a detailed description of the model tuning process in Appendix B for the case of CO${}_{2}$ time measurements.

**Figure 3:**
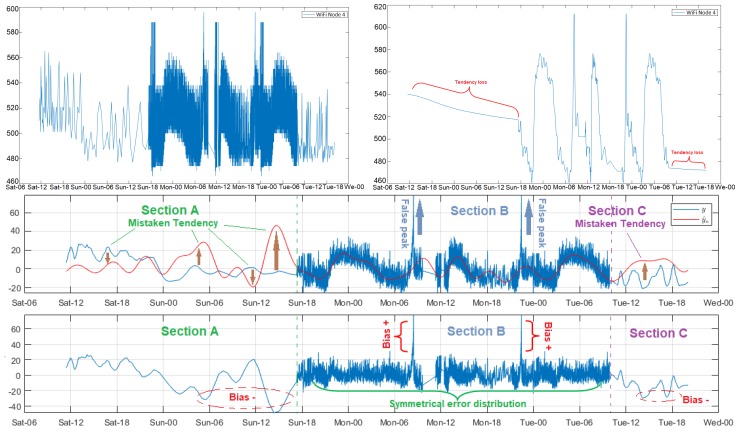
Modeling of CO${}_{2}$ signal in Sensor Node 4 of WiFi network, with Reg = 15 and for different sampling rates. From top to bottom: the original signal (**left**); the filtered signal with Ord = 100 (**right**); the rhythmometric analysis; and the residual time evolution with Ord = 4.

The acquisition time of several samples of the WiFi network nodes was increased from 8 s to approximately 8 min to analyze the incidence of the sampling rate decrease on the modeling. As an example, we considered again the volatile CO${}_{2}$ signal from Node 4, and the results obtained are shown in Figure 3. The selected filter order was considerably lower (Ord = 4) in relation to the Node 4 of DigiMesh network, because it was observed that, for low sample rates, and significantly increases of Ord, the original signal trend was lost. The sampling rate was lower for Sections A and C in the figure, the model was not well adjusted therein, and less accuracy was obtained for Reg values above 13, as evidenced through the amplitude mismatch between the observed and the estimated samples. In contrast, the adjustment was acceptable for Section B, where the sampling rate was higher, and in this context the residual magnitude was reduced and residuals remained symmetric throughout time, except for the bias resulting from the two distinctive peaks depicted in the graph. Accordingly, we conclude that, especially for the adjustment of fast transition variables, it is recommendable to use sampling rates in the order of some minutes, and then filtering the signal to discriminate against false peaks and noise.

Based on the model adjustment process of CO${}_{2}$ signal explained in Appendix B, we present the relevant results of the rhythmometric analysis the rest of environmental variables. In all cases, we applied filters to improve the signal quality, and Ord was defined according to the signal nature and its sampling rate. Considering the total length of the observed samples, the tool calculated 18 rhythms including $f_{o}$, where 1 corresponded to $f_{i}$, and 17 were in $f_{u}$. The number of significant spectral components $f_{u-sig}$, and $f_{i-sig}$ of the model were conditioned by the quality parameter Reg. Table 2 summarizes for each measured variables by the Sensor Nodes of the three WSNs the selected Ord, the recommended Reg, the number of significant frequencies, the sampling type, the histogram particular features such as distribution mode, the significant error range, and the relative frequency average of the two adjacent boxes to the error around ±1, as well as the matching limits defined by the tool for the Bland–Altman diagrams. The criteria for categorizing a model as acceptable were the following: temporal evolution of residuals with minimum and symmetric amplitude, statistical distribution of systematic error mostly close to Gaussian shape higher data concentration around zero, and concordance limits between observed and estimated samples symmetric and close to zero. The rhythmometric models were analyzed for different Reg values, and we concluded that the modeling was acceptable for a range where the results were reasonably close to the expected adjustment quality criteria.

**Figure 4:**
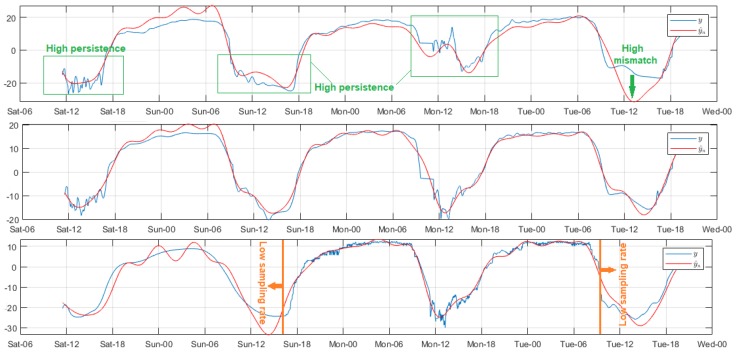
Representative modeled signals of air relative humidity. From top to bottom: Node 1 of DigiMesh network with Reg = 4, Node 2 of ZigBee network with Reg = 1, and Node 2 of WiFi network with Reg = 34.

Figure 4 shows three significant models of air relative humidity analysis, where in all cases the measurements tended to increase from approximately 18:00 to around 10:00 the next day. After that, the values decreased with high persistence until close to 18:00, when the variability pattern was repeated. The fluctuating behavior on measurements necessitated complicated model adjustment; the systematic errors were great in these sections; and the increase of Reg did not significantly improve the result quality. The mismatch was more pronounced during the signal final segment of Node 2 in the DigiMesh network. For the WiFi network, the error was lower for signal sections with low $f_{s}$. The modeling was more accurate for Node 2 of the ZigBee network, as shown by the narrow histogram error margins, and the reduced concordance limits of the scatter diagrams.

**Figure 5:**
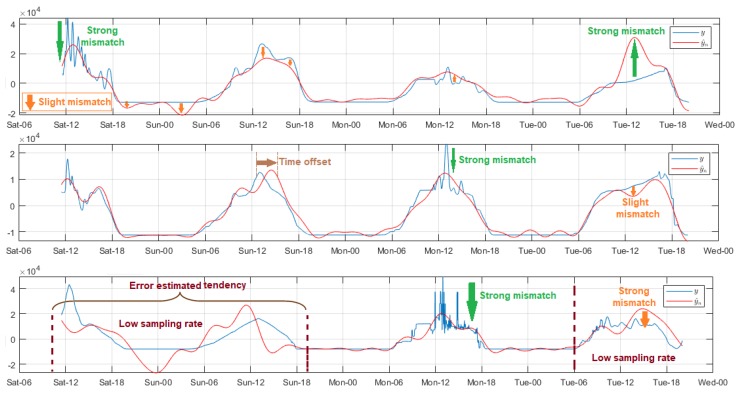
Representative modeled signals of luminosity. From top to bottom: Node 1 of DigiMesh network with Reg = 4, Node 2 of ZigBee network with Reg = 100, and Node 1 of WiFi network with Reg = 50.

Luminosity was a very noisy variable, therefore we applied a high order filter, except on the WiFi network because the sampling rate therein was different. Overall, the effective daily light hours were in the approximate range of 06:00. to 18:00 with maximum values between 10:00 and 15:00. As shown in Figure 5, the random variability, and the high amplitude of the peaks present in the signal affected the estimation quality, as evidenced by the pronounced biases of several time evolution sections of the residuals, and by the values over 2000 of the concordance limits in the Bland–Altman diagrams. The mismatch was evident, especially in the signal-end sections of Nodes 1 and 3 of the DigiMesh network, as well as in Node 2 of the ZigBee network, whereas, for Node 2 of the DigiMesh network, this was less noticeable. In Node 3 of the ZigBee network, the model was remarkably refined in the low luminosity sections with Reg>55, however this caused the mismatch of the remaining samples. In general terms, the quality parameters were affected negatively, so that a lower value was chosen. For the WiFi network, the negative impact of switching $f_{s}$ in Node 1 was noticeable, since the adjustment was not improved in signal sections with low rates, even for high Reg values. In contrast, the adjustments for Nodes 2 and 3 of this network were acceptable at both sample rates only for Reg∈(2,12), and outside this range the trend was lost, particularly in sections with low $f_{s}$. The models were more successful for Nodes 1 and 2 of the ZigBee and WiFi network, respectively, because most of the errors were distributed close to zero, and the concordance limits were significantly reduced in comparison with other nodes.

**Figure 6:**
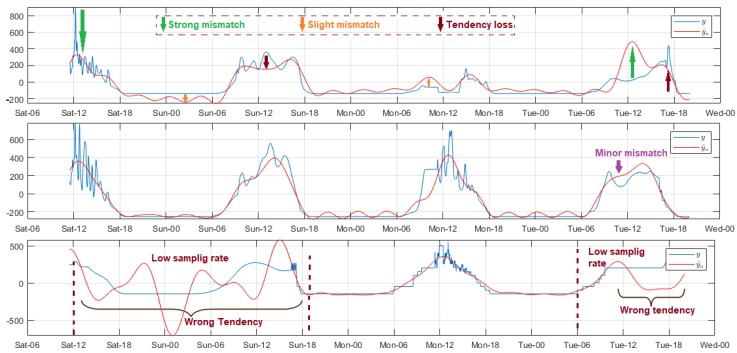
Representative modeled signals of solar radiation. From top to bottom: Node 1 of DigiMesh network with Reg = 1, Node 2 of ZigBee network with Reg = 300, and Node 2 of WiFi network with Reg = 2.

The solar radiation variations in time were similar to the luminosity, although the fluctuations were slowed-down in some signal intervals, and for this reason we selected a lower Ord filter to not modify the original signal trends. The adjustment features of each node are shown in Figure 6. In the initial measurements for Node 1 of the DigiMesh network, a considerable amplitude peak was recorded close to midday, which gradually affected the adjustment, causing a strong bias in the final section of the residual time evolution. During the experiment, Reg was increased from 1 to 400, and the results did not substantially improve. The progressive mismatch was lower in Node 2 of the ZigBee network. We concluded that, although the results show significant error range and the concordance limits were somewhat higher compared to the DigiMesh network node, the model quality was the best for this node, because the model adjustment was better, and the error frequency near zero increased dramatically. The modeling was less accurate for Node 2 of the WiFi network, since the adjustment tests were executed only for Reg=1, due to the $f_{s}$ change, whereas for other values the trend was lost, especially at low $f_{s}$. To more efficiently adjust the model of this variable, we suggest that the samples are acquired during major time intervals, considering that its behavior is similar to CO${}_{2}$, and the data trend differs greatly from one day to another.

**Figure 7:**
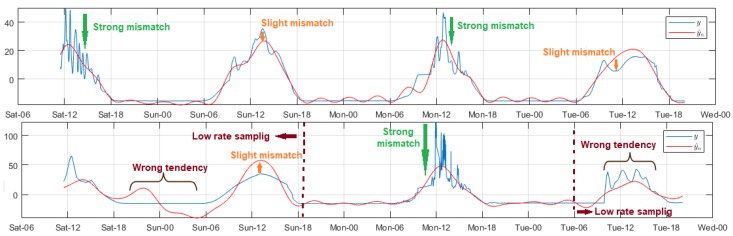
Representative modeled signals of UV radiation: (**Top**) Node 3 ZigBee network with Reg = 110; and (**Bottom**) Node 3 of WiFi network with Reg = 20.

The UV radiation was measured by specific nodes in each network. Figure 7 shows the relevant modeling results. The time behavior was very similar as explained for solar radiation, and we used the same order in the filters. However, the differences shown in the figure were that the progressive mismatch of the estimated samples were barely visible in the DigiMesh and ZigBee networks, and consequently the time variation ranges of the residuals decreased significantly. In the particular case of the WiFi network, the signal modeling at the low sampling rate was deficient, regardless of the Reg value, and also an accentuated radiation peak occurred for acquired samples at low $f_{s}$, producing a distinct positive bias in the residuals. Although Reg did not improve the model quality of this node, we included a shuffled value in Table 2. For this scenario, the significant error ranges of the histogram, as well as the Bland–Altman matching intervals, increased compared to the other two networks. It is relevant to note that the high frequency of near-zero errors on the histogram was not strictly related to a successful adjustment, because the residuals were very low for the signal sections with the largest samples. On this basis, we deduced that, for this variable, the model was more precise in Node 3 of the ZigBee network.

**Figure 8:**
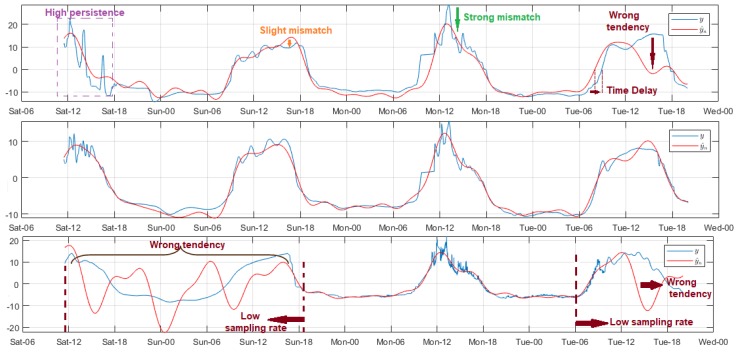
Representative modeled signals of air temperature. From top to bottom: Node 3 of DigiMesh network with Reg = 1, Node 2 of ZigBee network with Reg = 3, and Node 2 of WiFi network with Reg = 5.

Among all the studied environmental variables, the air temperature was one of the least volatile, and its fluctuations between samples were slow and with reduced amplitude, so we chose the same filter applied to the air relative humidity. The data variability trend was very similar in all nodes, and for all test days. The amplitude increased from about 08:00, and decreased from 17:00 approximately, and the air temperatures were the lowest in the dawn around 02:00–05:00. Figure 8 summarizes the representative models for this variable. For the DigiMesh network, the estimated signal of Node 1 allowed the original signal trend in almost the entire range, but with a slight amplitude offset with respect to the acquired samples. This mismatch was more noticeable in the final section. Considering that the errors and the concordance limits did not exceed 6 ${}^{\circ }$C and 2 ${}^{\circ }$C, respectively, we considered the model as acceptable. In Node 2, these offset particularities were also generated, although to a minor degree, and in general the model quality improved especially in the end-of-signal segment. In the particular case of Node 3, the initial measurements were persistent and unstable, therefore the residuals were greater, and this affected gradually the estimation of the other samples, and the mismatch was more evident in the last section, where the resulting modeling was inverse in comparison to the original signal. For the ZigBee network, the results for Node 1 were similar to those described for Node 1 of the DigiMesh network, although with a slight decrease in errors. The adjustment quality was very satisfactory throughout the signal even with minimums Reg values, and the model was more precise for Node 2. The measurements adjustment at the lowest sampling rate of the WiFi network nodes was very poor, being more evident in the initial lags. After experimentation in each node, we deduced that the regularization in several cases differed drastically even with minimal changes of Reg. After various experiments, we selected some values that improved the model, although poorly, and the results were as follows: Node 1 with Reg∈(4,62), Node 2 with Reg∈(4,8), and Node 3 with Reg∈(4,20). In all cases, the model trend was similar to the original signal; however, the time delay between the observed and the acquired samples was noticeable, and this mismatch was more evident for Nodes 1 and 2, as under these circumstances the resulting models were not capturing the true behavior of this variable. According to the explanation of previous paragraphs, we clarify that the apparently adequate quality parameters shown in Table 2 for these nodes were due to the adjustment errors being minor—several were nearly zero—for the signal sections whose samples volume were considerably superior, and not because the models were appropriate.

**Figure 9:**
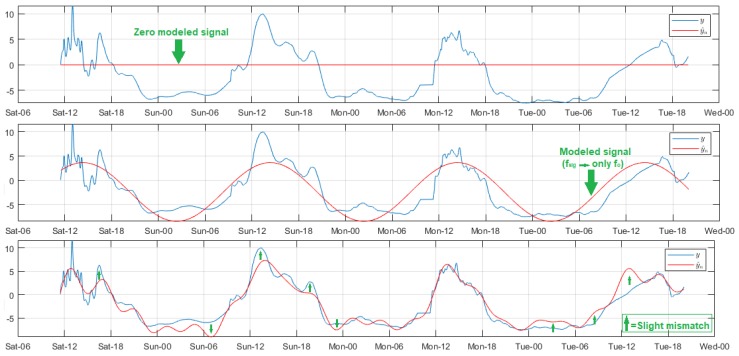
Representative modeled signals of wind speed. From top to bottom: Reg = 4, 5, and 12.

Figure 9 summarizes some relevant results of wind speed signal modeling. The measurements were acquired by Node 1 of the ZigBee network, and they were highly noisy. For this reason, we used a filter with Ord = 100. The signal trend was unchanged over time, however the amplitudes differed daily. Overall, the intensity increased from around 08:00, and peaked between 13:00 and 15:00, while the lowest values were registered from 24:00 to 08:00. The estimated samples were null for Reg values less than 5, due to the analysis tool discriminated all rhythmometric components. The modeled signal was perceptible from Reg = 5, however the results were quite poor as the analysis included only $f_{o}$. The model quality was greatly improved for Reg values above 11, except in specific signal segments where the mismatch was more prominent because of the daily amplitude variability of the original signal; this drawback was not solved, even with the increment of Reg. Based on these results, we concluded that model optimization for this variable requires more time stored data.

**Figure 10:**
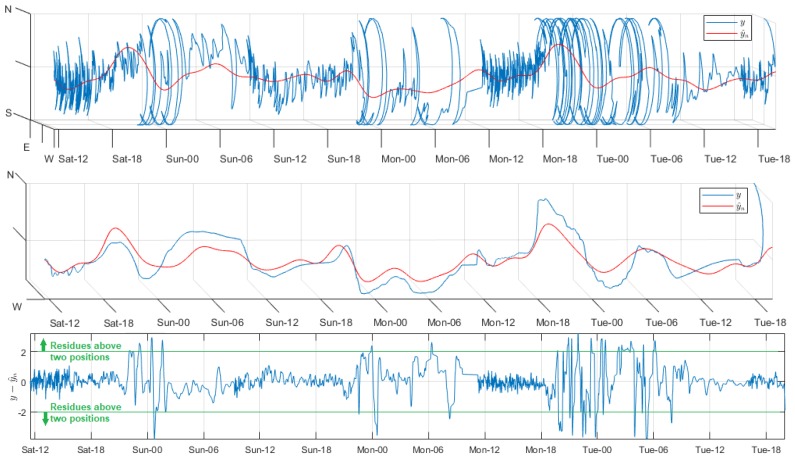

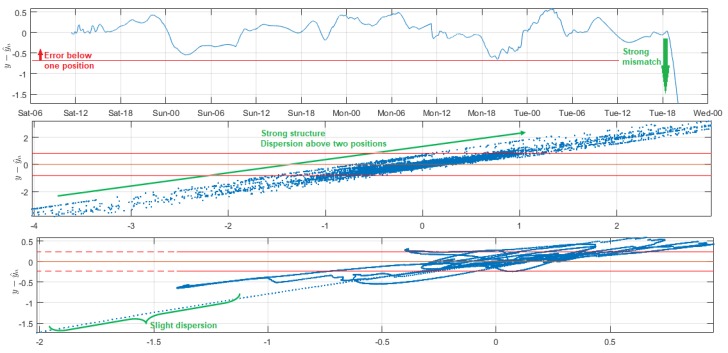
Representative rhythmometric analysis plots. From top to bottom: Non-filtered modeled signal (Reg = 2000), filtered modeled signal (Reg = 1700), residual time evolution without and with filter, and Bland–Altman diagram without and with filter.

The wind direction sensor was mounted on Node 1 of the ZigBee network. The acquired samples corresponded to positions in a circle, represented by integers between 1 and 16. Hence, an additional programming routine was developed for the data visualization and processing. Each position was transformed to cardinal points, and all of them were represented in a three-dimensional graph. During the experiment days, the wind was oriented to the northeast at different angles. In the analysis of this variable, two relevant particularities were presented that motivated the study both with and without filtering. First, the data type supplied by the sensor conditioned the modeling quality to residuals lower than one position, since a major error would mean an erroneous estimated direction. Secondly, the highly unpredictable and volatile behavior of the signal forced the signal filtering with an Ord value higher than the rest of the variables. Figure 10 shows the significant results of modeling with and without filtering. The unfiltered signal adjustment was quite poor, and the residuals over time exceeded the two positions, even with high Reg values. Even though the matching intervals of the Bland–Altman diagrams were less than one position, the dispersion was very strong and distant from these limits. The filtered signal model was substantially improved for Reg values above about 1700. The residuals were reduced to peaks lower than one position, and the Bland–Altman concordance limits were similar to the previous case. However, the dispersion inside them was robust and points out of the confidence intervals were considerably reduced, and not very distant from the expected values. The strong mismatch in the final segment of the filter-modeled signal was a consequence of the unpredictable behavior of this variable, and it was not corrected during the experimentation. To ensure a more accurate model, the sampling period should be much longer than the selected for this work.

### 5.3. Variable Benchmarking Among Sensor Nodes and Networks

This section describes the systematic inter-variable comparisons developed for the environmental monitoring of two tomato greenhouses. Several representative comparisons between pairs of signals of the same environmental time-series variable were carried out to scrutinize the dependence of the variables with the phenological stages of the tomato plants [59]. The comparisons of two different variables was tackled by time series representations together with scatterplots, and this was used to determine the relationship between the variables of greatest influence on the growth of the tomato crop. The comparisons of two series of the same variable was tackled by using Bland–Altman from two nearby nodes and two distant nodes, and they were carried out to establish the reliability of the measurement instruments. The comparisons between two nodes of the same network, as well as between similar nodes of different networks, were both made by means of scatters. As a general-view complement, the MI was scrutinized among all of them to analyze the incidence of measurements and their similarity depending on their location as well as the communication technology.

**Figure 11:**
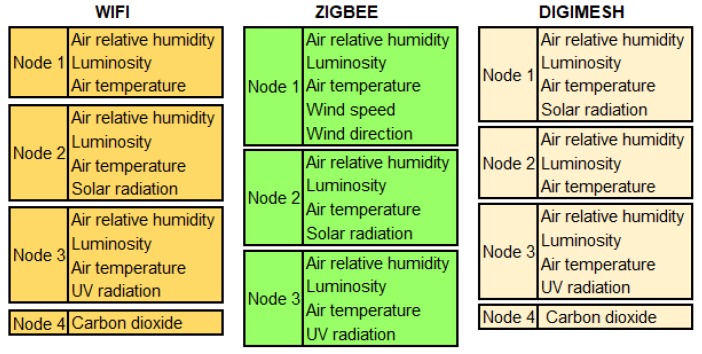
Distribution of the environmental variables for the sensor nodes of the three WSNs.

This analysis was performed in the three WSNs implemented in two greenhouses. The first one, Greenhouse A, consisted of the DigiMesh network, while the second one, Greenhouse B, consisted of the ZigBee and WiFi networks. Nodes 1–3 were installed at the contour of the greenhouses, and Node 4 was installed in the center, as shown in Figure 2. The distribution table presented in Figure 11 specifies the environmental variables of each sensor node.

**Figure 12:**
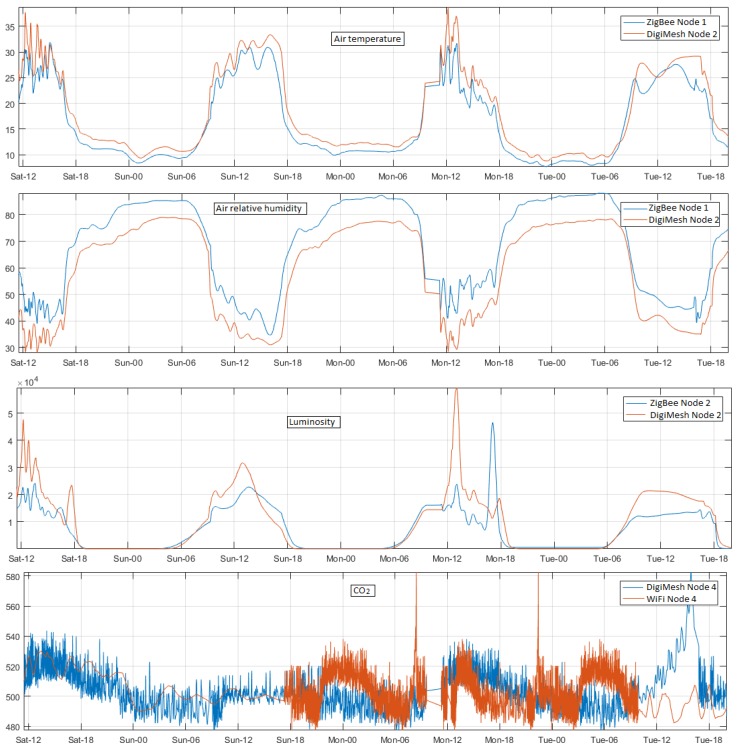
Temporal signals of the WSNs in different greenhouses.

The first comparison was made with the application of the filter Ord recommended in Table 2, so that this filtering eliminated the unwanted components improving the quality of the signals. Reduced CO${}_{2}$ is one of the primary factors affecting the quality of greenhouse tomatoes [60], and air temperature and air relative humidity are also considered as very important variables in the growth of these plants [61]. The luminosity can affect the tomato content of organic acids (citric and malic acids), sugar (glucose, fructose, and sucrose), solids that are insoluble in alcohol (proteins, celluloses, pectin, and polysaccharides), carotenes, and lipids, among others [62]. Figure 12 shows the changes in air temperature, air relative humidity, luminosity, and CO${}_{2}$ in different zones of the greenhouses. We verified that the differences among the measures of the variables were due to the phenology state of the tomato plants.

**Figure 13:**
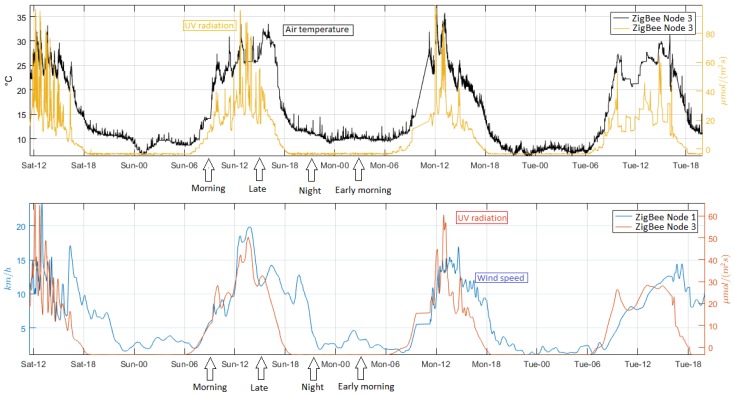

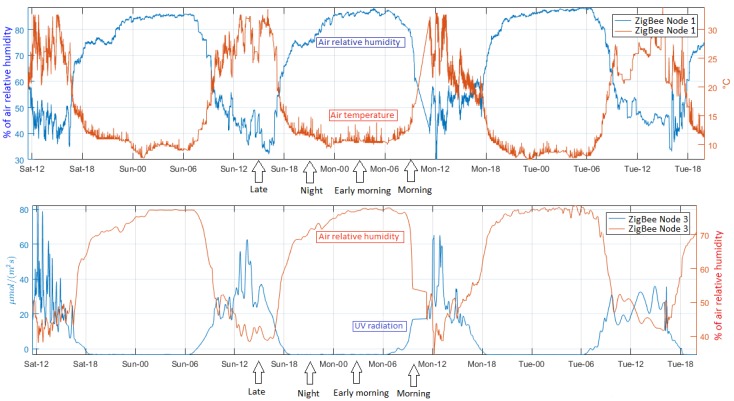
Temporal signals of the variables with direct and inverse relationship of the ZigBee network, from top to bottom.

**Figure 14:**
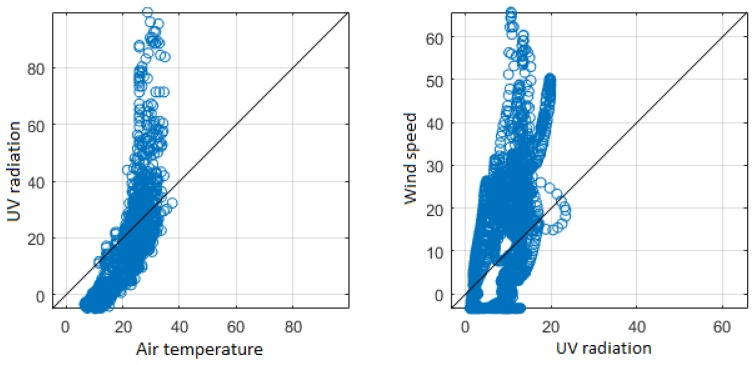

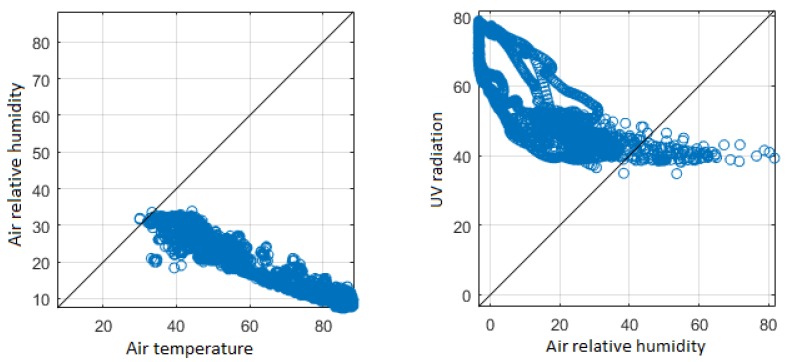
Scatter diagrams of the variables with direct and inverse relationship of the ZigBee network, from top to bottom.

For the analysis of the second comparison, the original signals were not filtered. This consideration was applied to observe the behavior of environmental variables over time. Figure 13 shows the temporal signals and the scatterplots to determine the changes and the relationship between the variables. Air relative humidity and air temperature are closely linked in the greenhouse [63]; a rapid variation occurs in the afternoon and slowly in the early morning, one variable increases while the other decreases slowly at night, and in the morning this change happens inversely. The plants grow under the influence of radiation (diurnal conditions) by performing the photosynthesis process [64]. The air temperature and UV radiation increase in the day and decrease in the night. There is another direct relationship between wind speed and UV radiation. In addition, Figure 14 presents the inverse correlation of air temperature and air relative humidity as well as the direct relation of UV radiation and air temperature. The continuous monitoring of environmental variables allows the analysis of climatic changes and hence to determine the optimal limits to prevent unwanted effects on growing tomatoes [12]. The analysis of the direct and inverse relationship of environmental variables allows the farmer to understand how greenhouse conditions influence crop growth, and to react to changes that are outside the permitted ranges to maximize productivity.

**Figure 15:**
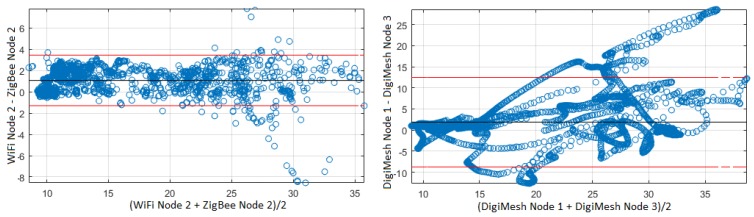
Bland–Altman plots of air temperature: (**left**) nearby sensor nodes; and (**right**) distant sensor nodes. This representation shows that the first case corresponds to measurements from very similar phenomena, whereas the second one corresponds to measurements from a complex-dynamics system which nevertheless are intrinsically related.

The third comparison was developed with the use of the filter to eliminate scattered values, and, with this stage, the width of the confidence interval was slightly reduced. The Bland–Altman plots for the air temperature data are shown in Figure 15. The *X*-axis shows the average of the two signals. The red lines represent the 95% confidence limits of the differences between the two networks. A regression line is shown that indicates that the mean differences between the networks are nearly zero. The difference of means between the signals of air temperature of Node 2 of the WiFi and ZigBee networks is 4.753, with agreement limits of −1.303 and 3.45. The agreement limits are narrow due to the strong correlation and the nearby location of the sensor nodes. The difference of means between the air temperature signals of Node 1 and 3 of the DigiMesh network is 21.44, with limits of agreement −8.744 and 12.5. The agreement limits are wide due to the weak correlation and the distant location of the sensor nodes. Accordingly, the reproducibility of the measurements is strongly related to the location of the sensor nodes.

**Figure 16:**
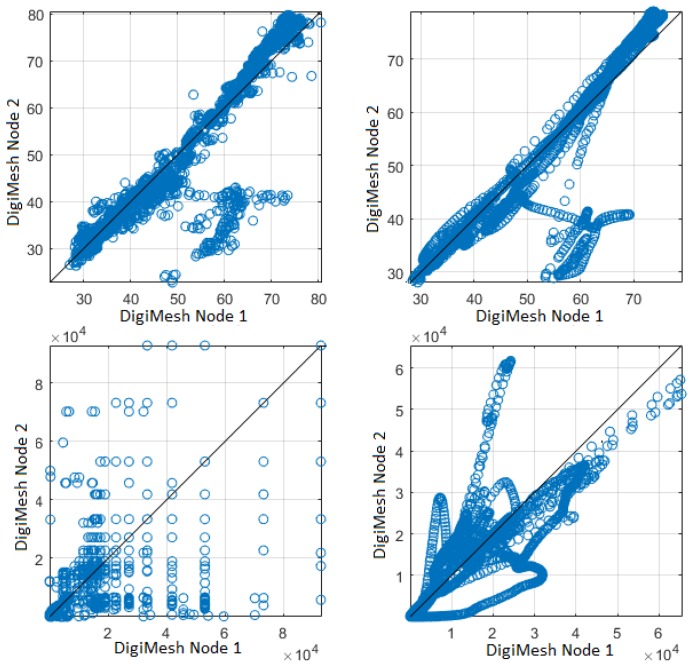
Scatterplots between two sensor nodes in the DigiMesh network: (**top**) air relative humidity, showing that the moderate noise does not mask the existence of two kinds of states, one for similar measurements, and another for different inter-node measurements; (**bottom**) luminosity; (**left**) not filtered; and (**right**) filtered, showing, in this case, the strong structuring effect of filtering on both signals, and the differences in their measurements, which are not evident in the unfiltered version.

**Figure 17:**
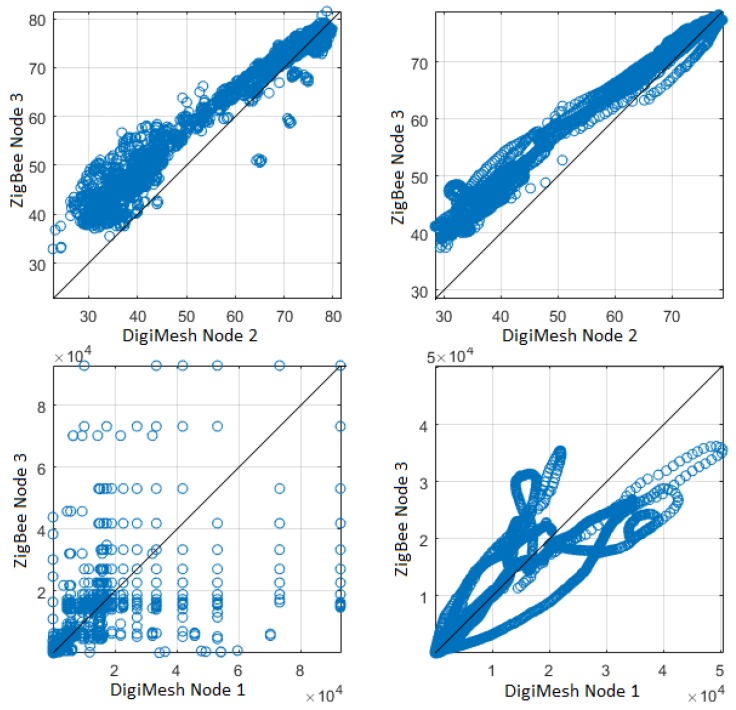
Scatterplots between two Sensor Nodes in the DigiMesh and ZigBee networks: (**top**) air relative humidity, showing the strong similarity and the patent bias in both cases; (**bottom**) luminosity; (**left**) not filtered; and (**right**) filtered.

In the fourth comparison, the original signals were filtered so that scattered values were excluded. The filter order was selected for a better correlation among the sensor nodes. Figure 16 shows the scatter plots of relative air relative humidity and luminosity of Node 1 and 2 for the DigiMesh network, respectively. We observed that there is a moderate positive linear correlation between Nodes 1 and 2 of air relative humidity. In addition, Nodes 1 and 2 of luminosity had a positive linear correlation in the initial values. However, these correlations were not strong between the variables, because the sensor nodes were located in the right corners of Greenhouse A. Figure 17 shows the scatterplots between two different network sensor nodes for the air relative humidity and luminosity signals. Sensor Nodes 2 in DigiMesh and 3 in ZigBee for air relative humidity exhibited a positive linear correlation in the final values. Sensor Nodes 1 in DigiMesh and 3 in ZigBee for luminosity exhibited a positive linear correlation in the initial values. The WSNs were implemented in two different greenhouses, which explains the weak linear correlation between these variables.

**Figure 18:**
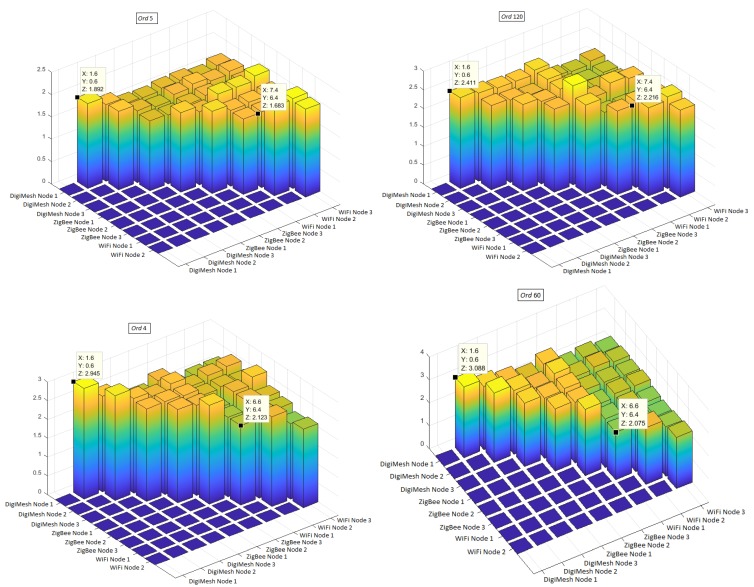
MI comparison with change of Ord between all different sensor nodes of the three WSNs for luminosity and air relative humidity. from top to bottom.

The correlation analysis is strongly limited by its linear nature. Therefore, the variables can be related in an arbitrary way, so that it is necessary to use more general techniques to analyze their degree of dependence [65]. As a complement to the correlation, comparative analysis was made with the scatterplots. The MI was calculated among the variables of all the sensor nodes, so that we can easily appreciate which variables are more associated. The MI matrix shows the similarities between variables. MI was not estimated for the variables with themselves, so that the diagonal of the matrix was depicted as zero. The upper triangular submatrix has been preserved to facilitate its visualization. The lower triangular and the diagonal was plotted as zero, and also the last row (Node 3 of the WiFi network) was all zero; nevertheless, all the comparisons of the WiFi nodes were shown as an example in Figure 18. The filter Ord caused a higher incidence in the MI values for volatile variables such as luminosity, so with Ord of 5 it was 1.892 bits and with Ord of 120 it was 2.411 bits. Meanwhile, in slow variables such as relative humidity, the values of MI change slightly, so with Ord of 4 it was 2.945 bits and with Ord of 60 it was 3.088 bits. For the MI analysis, we recorded the values of MI with and without filtering, as shown in Table 3 and Table 4. The application of the filter caused a minimum change in the MI values. A decrease occurred in the comparison between two sensor nodes of the same WSN for the variables of air relative humidity and air temperature of the WiFi network. In addition, when comparing the sensor nodes of different WSNs, the MI values decreased for the CO${}_{2}$ and UV radiation of the pairs between DigiMesh-ZigBee and ZigBee-WiFi.

Table 3 shows the MI values compared between two sensor nodes for air relative humidity, luminosity and the air temperature of the same WSN. The value of MI was significantly high in the DigiMesh network, when Nodes 1 and 2 were considered for air relative humidity, confirming the highest correlation as shown in Figure 16. MI values were high in the ZigBee network between Nodes 1 and 3 for luminosity, and Nodes 2 and 3 for air temperature. The value of MI was lower in the WiFi network, when Nodes 2 and 3 were compared for the air temperature. The MI values were low in WiFi network between Nodes 1 and 2 for air relative humidity, and Nodes 2 and 3 for luminosity. Therefore, the location of sensor nodes did not affect the degree of similarity of the signals for the DigiMesh and ZigBee networks. Table 4 shows the MI values compared between two sensor nodes of different WSNs. The MI value was high for the air relative humidity (2.807 bits), luminosity (2.550 bits), air temperature (2.761 bits), and solar radiation (4.458 bits) between the sensor nodes of the DigiMesh and ZigBee network. The value of MI was low for air relative humidity (2.016 bits), luminosity (1.571 bits), air temperature (1.534 bits), and solar radiation (3.462 bits) between the sensor nodes of the DigiMesh and WiFi network. Therefore, the type of communication technology affected the degree of similarity for the comparison between DigiMesh and WiFi networks. There was a significantly lower MI value in the CO${}_{2}$ variable, because it is a faster variable than the other ones.

## 6. Discussion and Conclusions

We studied the signal dynamics of environmental variables CO${}_{2}$, air relative humidity, luminosity, solar radiation, UV radiation, air temperature, wind speed, and wind direction, in two tomato greenhouses located in the Ecuadorian region of the Andes. The measurements were acquired during four days through several Sensor Nodes of three WSNs; two networks used ZigBee technology (star topology, and mesh denoted as DigiMesh), and a WiFi technology (access point topology). The sampling rate was constant for the ZigBee and DigiMesh networks at approximately 0.333 samples per second, and variable for the WiFi network at around 0.125 and 2.083 ×$10^{-3}$ samples per second. The data were stored in databases compatible with Matlab${}^{\mathrm{TM}}$.

The dynamics were analyzed from two viewpoints. In the first one, we adjusted the parametric model of each variable considering its cyclo-seasonality by means of rhythmometric analysis, and supported by the bootstrap method for the selection of significant spectral components. The models were validated by the residual time evolution and statistical representation (Bland–Altman diagrams and histograms). In the second instance, we scrutinized the dependence between measurement pairs by considering three systematic comparisons. We initially analyzed the correlation between the most influential variables in greenhouse crop growth through time series and scatterplots. Next, we compared the measurements of the same variable in different nodes to evaluate the accuracy of the measurement instruments by means of Bland–Altman graphs. Finally, we compared the measurements between nodes of the same network and of different networks with MI to define the incidence of the nodes location, and of the communication technology type with respect to the variable association.

The filtering incidence in the results was analyzed in all test scenarios. For the ZigBee and DigiMesh networks, the Ord parameter was tuned based on the noise, speed changes of each signal, and the permanence of the original trend. The wind direction was the most volatile signal, so we used a filtering with larger Ord. The change rate was slightly reduced in the luminosity, CO${}_{2}$, and wind speed, therefore a filtering with slightly lower Ord was required. The air relative humidity, air temperature, solar radiation, and UV radiation signals were less noisy and slow, and for this reason an Ord value was assigned being considerably lower with respect to the other variables. Regardless of the nature of the signals of the WiFi network, the variable sampling rate limited the filtering at Ord values below 6, to prevent the distortion of the original signal trend, especially in segments with low $f_{s}$.

To adjust the models of each environmental variable, we developed a selection process for the quality parameters Reg and Ord, to ensure that the estimated samples kept the original trend, and if possible were situated in the middle of the samples and have reduced bias. The models were considered acceptable when the amplitudes of the temporal evolution of the residuals were low and symmetric, if the statistical distribution of the systematic error was approximately Gaussian with a high concentration of data around zero, and if the limits of agreement between the observed and estimated samples were small and nearly symmetrical. We verified that the filtering inclusion in all variables improved the model quality, this becoming more noticeable in the volatile variables, especially in the wind direction.

Through the experimentation phase, we identified two factors that significantly affected the accuracy of the model adjustment. The first was the instability or persistence in some signal sections, particularly in the air relative humidity of Node 1 of the DigiMesh network. A further aspect was the high variability of the samples amplitudes between days, combined with the presence of significant peaks or drops in the signal, this being the most common cause of mismatch, and it was present in the CO${}_{2}$, solar radiation, UV radiation, wind speed, and wind direction measurements. Based on this experience, we concluded that the data acquisition period for these variables should be longer than the one used in this work, to optimize the learning of the analysis tool, and to increase the modeling accuracy on a long-term basis. In addition, we deduced that these factors mostly affected the DigiMesh network nodes, and that the models were more accurate for the signals acquired by the ZigBee network nodes, particularly in Node 1 for brightness; Node 2 for air relative humidity, air temperature, and solar radiation; and Node 3 for UV radiation. The wind speed was measured only on Node 1 of the ZigBee network, and its modeling cannot be compared with the other technologies; however, the estimation was acceptable, except in some specific signal sections where the mismatch was more pronounced because of its varying behavior over days. The slowest signal was the air temperature, and the daily measurements were very similar for the nodes of the three networks, so we concluded that the modeling of this variable was the most accurate, regardless of the nodes location and of the communication technology (ZigBee or DigiMesh). The adjustment of the WiFi network models was very deficient for the signal segments with low sampling rate, especially in the noisy and fast transition variables, therefore we recommend the measurements acquisition at high sample rates to improve the quality of the models.

The modeling analysis and the filtering effects in the wind direction were individualized, because, in contrast with the other variables, the data acquired were integer numbers that oscillated between 1 and 16 that correspond to positions (cardinal points). Therefore, we considered a suitable model only if the residuals were not above one position, otherwise the estimated samples were completely deviated with respect to the actual direction. The signal adjustment without filter was very deficient, because the residuals time evolution exceeded two positions even for high values of Reg. The model was markedly improved for the filter signal using Reg values higher than about 1700; however, a strong mismatch occurred in the final segment of the estimated signal as a consequence of the non-periodic behavior of this variable.

The results of the comparison between different variables revealed that air relative humidity was inversely correlated to air temperature, solar radiation, and wind speed, whereas UV radiation was directly associated with respect to air temperature and wind speed. Regarding the comparisons of two temporal series of the same variable, the correlations were strong for the nearby nodes and weak for the distant nodes, therefore, the reproducibility of the measurements was related to the location of the sensor nodes. According to the statistical cross-references of the variables among the sensor nodes of the same network, the air temperature data for the WiFi network nodes were the least correlated. The bits resulting from MI also evidenced less dependence; hence, the nodes location affected the degree of similarity of the signals for the WiFi network. Moreover, the cross-information study of variables between nodes of different networks revealed that the luminosity, air temperature, and CO${}_{2}$ measurements of the nodes of the DigiMesh and WiFi networks were scarcely correlated. The MI values also indicated less dependence between the sensor nodes for these variables, thus we concluded that the communication technology type affected the analogously to the acquired signals of the DigiMesh and WiFi networks. The addition of the filter to the benchmarking development reinforced the data correlation of the scatterplots, partially narrowed the Bland–Altman confidence intervals, and improved the association between the variables in most comparisons as evidenced in the values showed in the MI tables.

Much more information could be retrieved for describing the environmental variable dynamics that have been addressed here. Nevertheless, in the present study, we constrained ourselves to analyze their short-term and middle-term dynamics, as, in doing so, we were able, for instance, to analyze with detail the impact of sampling rate starting from high rates. The extension of the analysis to longer periods and to different climate conditions is strongly desirable, especially in terms of frost conditions, which is the main problem for the tomato cultivation in Ecuador. This research can also be complemented through the design and implementation of a frost early warning system, described as a climatic phenomenon that causes serious crop losses, principally in the Ecuadorian Andes. The data will be acquired at a steady sampling rate for a time span not shorter than one year, through a wireless network of high-precision weather stations located in strategic areas. Based on the modeling and statistical analysis tools described in this paper, we propose the development of an algorithm to predict the behavior of the most influential variables when frost occurs. The early diffusion of possible frost through social networks and mobile phones could contribute to the timely execution of corrective actions to avoid crop damage and economic losses.

## Figures and Tables

**Table 1:** State of the art of relevant works in the WSN literature related to environmental variable data analysis.

Work	Case Study Variables	Technical Contribution
Aquino et al. (2011) [37]	Air temperature, air relative humidity, soil temperature, and moisture.	Development of a new platform for wireless sensor networks, with a modified version of the routing algorithm LORA_CBF to precision agriculture.
Keshtgary et al. (2012) [38]	Water-level, gate position, rainfall, and soil moisture.	Performance metrics (delay, throughput and load) of WSN for precision agriculture using grid and random topology.
El-Kader et al. (2013) [39]	Soil moisture, air elative humidity, air temperature, pH, and luminosity.	Precision farming solution for potato crop in Egypt using WSN.
Mansouri et al. (2013) [40]	Soil moisture.	Comparison of estimating methods using three different filters (Variational, Kalman and Extended Kalman) for state variables of crops.
Kodali et al. (2016) [41]	Soil moisture.	Water stress monitoring during dry season on coffee crops in India using WSN irrigation management.
Ferrández et al. (2016) [42]	Luminosity, water PH level, atmospheric humidity, and electric conductivity.	Heterogeneous and scalable platform based on Ubiquitous Sensor Networks (USN) and Internet of Things (IoT) paradigms for crop automation.
Piamonte et al. (2017) [43]	PH, humidity, air temperature and luminosity.	Analysis of environmental variables of influence on African palm cultivation using big data tools
Ponce et al. (2017) [44]	Soil moisture, air relative humidity, air temperature, luminosity level, and CO${}_{2}$	Greenhouse WSN data analysis using data mining.
García Ruiz et al. (2018) [45]	Air temperature.	Indoor greenhouse air temperature collection using WSN
Caicedo et al. (2018) [46]	Soil moisture, and soil temperature.	Development of a prototype for monitoring of agronomic variables in cassava crops, and modeling to determine the location nodes.
Lee (2013) [47]	Air temperature, and humidity.	Design of an agricultural production system based on IoT for predicting the growth and quantity of crop production.
Chapman et al. (2018) [48]	Air relative humidity, PH, and air temperature.	Design of Bayesian networks to predict the performance functions of three commercial oil palm farms.

**Table 2:** Relevant parameters for model adjustment. For each network, node and sensor, the filtering order (Ord), the regularization parameter (Reg), and the number of significant infradian and ultradian components ($f_{i}\_sig$ and $f_{u}\_sig$) are indicated. Error range, average relative frequency and mode of the residual histograms are indicated to summarize the residual analysis. The matching limits for the data and the model are also provided, as well as the qualitative description of the relevant features of the residual histograms.

Variable (Measuring Unit)	Network	Sensor Node	Ord	Reg	$f_{i}\_\mathrm{sig}$	$f_{u}\_\mathrm{sig}$	fs	Error Range	Average Relative Frequency	Matching Limits	Histogram Distribution
**CO${}_{2}$ (ppm)**	**DigiMesh**	4	100	33	1	16	CSR	−6, +5	778	−2.78, +2.78	GA
	**WiFi**	4	4	13	0	15	VSR	−15, +15	884	−8.64, +8.65	G
**Air relative humidity (% RH)**	**DigiMesh**	1	50	4	1	16	CSR	−10, +8.5	449	−3.91, +3.91	NG, U, LB
		2	50	5	1	16	CSR	−7.5, +6.5	929	−2.62, +2.63	NG, U, LB
		3	50	1	1	16	CSR	−7.5, +6.5	906	−2.73, +2.73	NG, U, LB
	**ZigBee**	1	50	5	1	16	CSR	−9.5, +8.5	685	−3.12, +3.13	NG, U, LB
		2	50	1	1	16	CSR	−5.7, +4.2	752	−1.67, +1.67	NG, U, LB
		3	50	32	1	16	CSR	−6.6, +5.4	501	−1.94, +1.98	NG, U, LB
	**WiFi**	1	6	6	1	16	VSR	−6.2, +5.6	1909	−1.52, +1.52	NG, U, RB
		2	6	34	1	11	VSR	−6.6, +3.9	2479	−1.90, +1.89	GA
		3	6	25	1	10	VSR	−9, +4.5	1426	−2.11, +2.10	NG, U, LB
**Luminosity (Lux)**	**DigiMesh**	1	120	110	1	13	CSR	−1.1 × 10${}^{4}$, +10${}^{4}$	1416	−4859, +4787	NG, U, RB
		2	120	100	1	15	CSR	−1.5 × 10${}^{4}$, +1.9 × 10${}^{4}$	1345	−4987, +4899	NG, M, LB
		3	120	10	1	16	CSR	−1.05 × 10${}^{4}$, +10${}^{4}$	1345	−3674, +3667	NG, M, RB
	**ZigBee**	1	120	100	1	15	CSR	−0.5 × 10${}^{4}$, +0.8 × 10${}^{4}$	1038	−2780, +2707	NG, M, LB
		2	120	420	1	14	CSR	−1.05 × 10${}^{4}$, +10${}^{4}$	800	−4374, +4176	NG, M, RB
		3	120	40	1	16	CSR	−1.05 × 10${}^{4}$, +1.65 × 10${}^{4}$	1151	−3844, +3813	NG, U, RB
	**WiFi**	1	5	50	0	8	VSR	−1.5 × 10^4^, +1.65 × 10${}^{4}$	1742	−4516, +4511	NG, M, LB
		2	5	12	1	12	VSR	−0.51 × 10${}^{4}$, +0.93 × 10${}^{4}$	2899	−2339, +2244	NG, U, LB
		3	5	12	0	7	VSR	−0.85 × 10${}^{4}$, +1.15 × 10${}^{4}$	826	−3863, +3863	NG, M, LB
**Solar radiation (nm)**	**DigiMesh**	1	60	1	1	15	CSR	−160, +210	1082	−83.65, +83.64	NG, M, RB
	**ZigBee**	2	60	300	1	15	CSR	−240, +260	2038	−84.38, +97.8	NG, U, LB
	**WiFi**	2	4	1	0	5	VSR	−200, +260	639	−77.45, 77.15	NG, M, LB
**UV radiation (nm)**	**DigiMesh**	3	60	42	1	15	CSR	−15, +18	1569	−5.92, +5.78	NG, U, RB
	**ZigBee**	3	60	110	1	16	CSR	−13, +18	1753	−5.58, +5.46	NG, U, LB
	**WiFi**	3	4	20	1	7	VSR	−22, +25	1980	−10 × 10${}^{-7}$, +10.06	NG, M, RB
**Air temperature (${}^{\circ }$C)**	**DigiMesh**	1	50	14	1	15	CSR	−4.2, +5.7	730	−2, +2	NG, M, RG
		2	50	5	1	16	CSR	−4.2, +5.1	728	−1.86, +1.86	NG, M, RB
		3	50	7	1	16	CSR	−10, +12	611	−3.68, +3.68	NG, M, LB
	**ZigBee**	1	50	6	1	13	CSR	−5.4, +5.7	693	−1.87, +1.87	NG, U, LB
		2	50	3	1	15	CSR	−4, +4.6	925	−1.34, +1.34	NG, U, LB
		3	50	17	1	15	CSR	−4.2, +5	616	1.48, +1.48	NG, M, LB
	**WiFi**	1	4	4	0	9	VSR	−7.8, +7.5	978	−2.3, +2.3	NG, M, RB
		2	4	8	1	11	VSR	−4.2, +5.6	2546	−1.5, +1.5	NG, U, RG
		3	4	20	1	12	VSR	−3.2, +4.8	3039	−1.32, +1.33	NG, U, LB
**Wind speed (km/h)**	**ZigBee**	1	100	12	1	13	CSR	−2.8, +3.6	682	−1.36, +1.35	NG, M, LB
**Wind direction (Positions)**	**ZigBee**	1	200	1700	1	16	CSR	−0.66, +0.54	989	−0.23, +0.24	NG, M, RB

CSR, Constant Sampling Rate; VSR, Variable Sampling Rate; GA, Approximate Gaussian; NG, Non-Gaussian; G, Gaussian; M, Multi-modal; LB, Left Bias; RB, Right Bias.

**Table 3:** MI results compared between two sensor nodes of the same WSN.

Variable	MI (bits)	DigiMesh		ZigBee		WiFi	
		Unfiltered	Filtered	Unfiltered	Filtered	Unfiltered	Filtered
**Air relative humidity**	Node 1 - Node 2	2.866	3.088	2.442	2.718	2.373	2.392
	Node 1 - Node 3	2.489	2.756	2.361	2.639	1.993	2.018
	Node 2 - Node 3	2.724	3.025	2.580	2.880	2.083	2.090
**Luminosity**	Node 1 - Node 2	1.832	2.431	1.754	2.626	1.774	1.971
	Node 1 - Node 3	1.662	2.433	1.586	2.986	1.810	2.015
	Node 2 - Node 3	1.686	2.304	1.836	2.737	1.807	1.935
**Air temperature**	Node 1 - Node 2	2.203	2.626	2.233	2.640	1.581	1.613
	Node 1 - Node 3	1.795	2.148	2.275	2.621	1.801	1.860
	Node 2 - Node 3	1.808	2.129	2.420	2.984	1.527	1.562

**Table 4:** MI results compared between two sensor nodes of different WSNs.

Variable	MI (bits)	DigiMesh - WiFi		DigiMesh - ZigBee		ZigBee - WiFi	
		Unfiltered	Filtered	Unfiltered	Filtered	Unfiltered	Filtered
**Air relative humidity**	Node 1 - Node 1	2.011	2.016	2.234	2.642	2.112	2.112
	Node 2 - Node 2	2.347	2.373	2.357	2.558	2.342	2.373
	Node 3 - Node 3	2.185	2.245	2.385	2.720	2.167	2.196
	Node 1 - Node 2	2.136	2.133	2.150	2.428	2.354	2.384
	Node 1 - Node 3	2.077	2.142	2.352	2.702	2.560	2.5885
	Node 2 - Node 3	2.360	2.420	2.486	2.807	2.180	2.267
**Luminosity**	Node 1 - Node 1	1.560	1.633	1.556	2.405	1.836	1.883
	Node 2 - Node 2	1.542	1.617	1.495	2.198	1.629	1.711
	Node 3 - Node 3	1.611	1.668	1.566	2.454	1.674	1.765
	Node 1 - Node 2	1.489	1.571	1.588	2.367	1.754	1.856
	Node 1 - Node 3	1.599	1.655	1.590	2.55	1.894	1.959
	Node 2 - Node 3	1.592	1.681	1.595	2.454	1.701	1.754
**Air temperature**	Node 1 - Node 1	1.726	1.791	1.976	2.541	1.794	1.860
	Node 2 - Node 2	1.689	1.761	2.133	2.699	2.046	2.119
	Node 3 - Node 3	1.449	1.534	1.715	1.993	1.644	1.733
	Node 1 - Node 2	1.699	1.832	2.030	2.444	1.805	1.895
	Node 1 - Node 3	1.550	1.599	2.107	2.53	1.632	1.705
	Node 2 - Node 3	1.579	1.631	2.209	2.761	1.623	1.654
**Solar radiation**	Node 1 - Node 2	3.057	3.462	4.304	4.458	3.384	3.878
**UV radiation**	Node 3 - Node 3	2.412	3.138	2.033	1.844	1.918	1.912
**CO${}_{2}$**	Node 4 - Node 4	0.766	0.637	—–	—–	—–	—–

**Figure A1:**
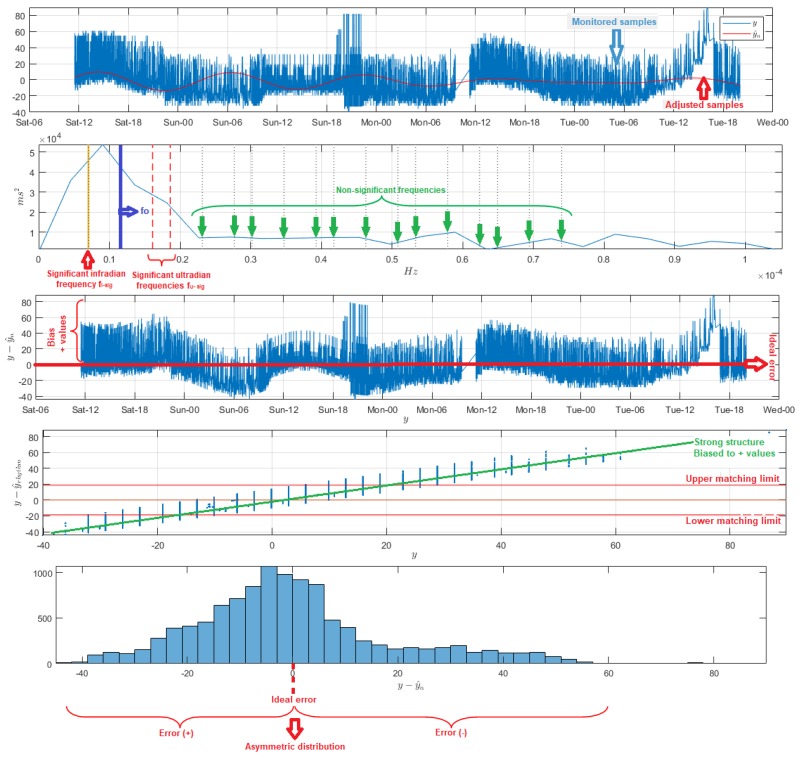
Modeling the CO${}_{2}$ signal at Sensor Node 4 of DigiMesh network (Ord = 0, and Reg = 0). From top to bottom: Rhythmometric analysis, frequency components in the FFT, residual time evolution, Bland–Altman diagram, and residual histogram.

**Figure A2:**
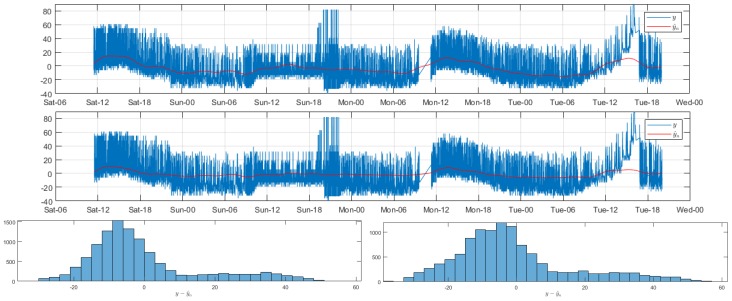
Modeling the CO${}_{2}$ signal in Sensor Node 4 of DigiMesh network (Ord = 0). From top to bottom: Rhythmometric analysis with Reg = 10 and Reg = 10,000, and histograms for their corresponding residuals (left and right).

**Figure A3:**
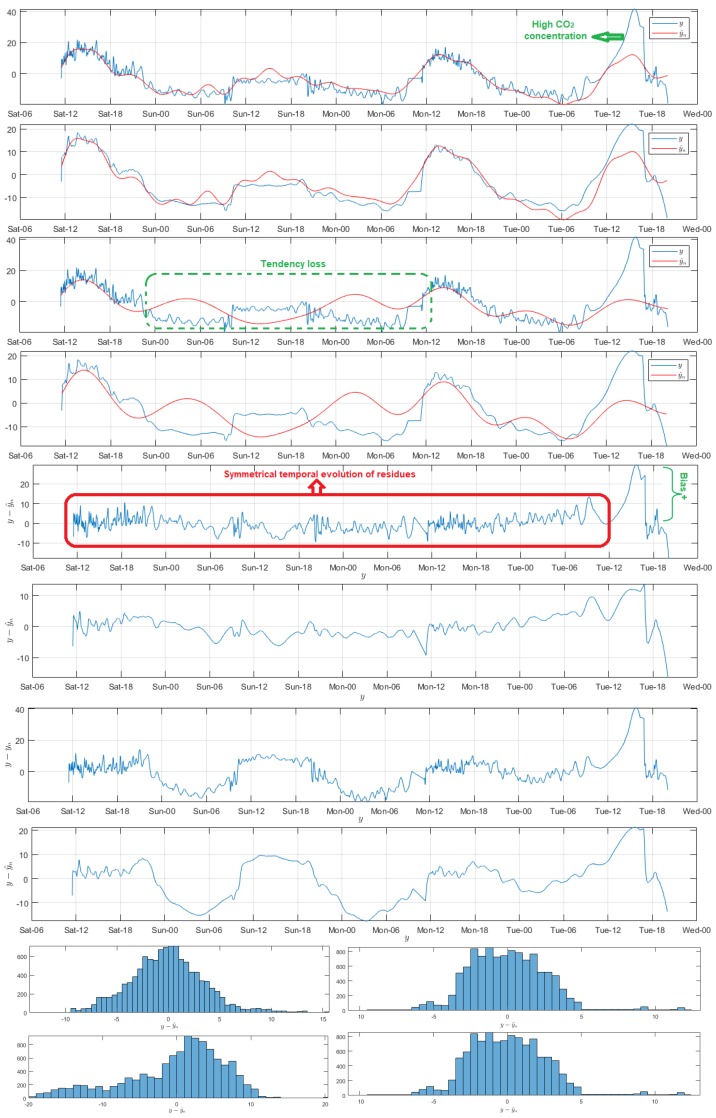
Modeling of CO${}_{2}$ signal from Sensor Node 4 of DigiMesh network. From top to bottom: Rhythmometric analysis with Reg = 15 and Ord = 30; Reg = 15 and Ord = 100; Reg = 40 and Ord = 30; Reg = 40 and Ord = 100; Residual time evolution with Reg = 15 and Ord = 30; Reg = 15 and Ord = 100; Reg = 40 and Ord = 30; and Reg = 40 and Ord = 100; Histograms with Reg = 15 and Ord = 30 (**left**); and Reg = 15 and Ord = 100 (**right**); and Histograms with Reg = 40 and Ord = 30 (left); and Reg = 40 and Ord = 100 (right).

**Figure A4:**
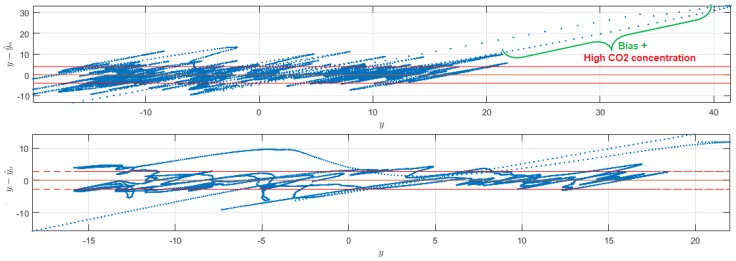

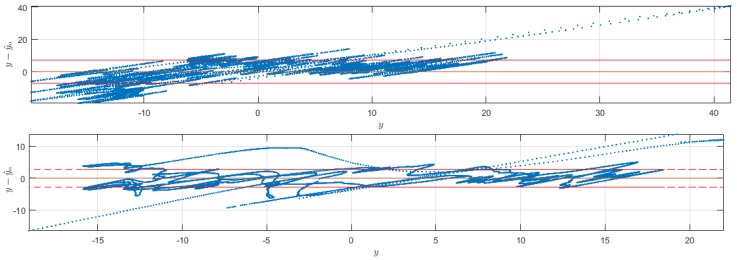
Modeling the CO${}_{2}$ signal in Sensor Node 4 of DigiMesh network. From top to bottom: Bland–Altman plots with Reg = 15 and Ord = 30; Reg = 15 and Ord = 100; Reg = 40 and Ord = 30,; and Reg = 40 and Ord = 100.

## Appendix A. Mathematical Notation and Detailed Equations

Here, the mathematical notation is detailed and the statistical methods are explained in more detail for the interested reader.

*Rhythmometric Analysis and Bootstrap Order Selection.* The single Cosinor model signal is denoted by $y_{n}$, and it is determined by the following equation,

(A1) $$y_{n}=M+A_{o}\cos\left(2\pi f_{o}t_{n}+\phi_{0}\right)+e_{n}$$

where the data samples are defined for n=1,…,N, where *N* is the signal length; *M* (for MESOR, from Midline Statistic of Rhythm) is the rhythm estimated average; $A_{o}$ is the adjusted cosine amplitude, and indicates the half of the extent of the predictable variation in a cycle; $f_{o}$ is the fundamental frequency, often chosen to be the circadian component; $\phi_{0}$ (also called acrophase) is the time interval from the starting point to the maximum of the cosine curve adjusted to the data; and $e_{n}$ is a random variable that represents the error calculated for each time instant and measured by the model residuals. To analyze the model error, it is often assumed that they are independently and normally distributed. The model residuals are calculated by the difference between the observed sample $y_{n}$ and the value provided by an estimated regression model, denoted by ${\hat{y}}_{n}$. The regression parameters are determined by using the well-known least squares (LS) method [66].

The multiple Cosinor regression model can include the modeling of additional components to the circadian ones. In chronobiology problems, where the seasonalities are often among the strongest temporal driving effects, several ultradian ($f_{u}$) and infradian ($f_{i}$) components are often included in the model. Considering the moderate time length of the acquired samples in this work (up to four days), we included several infradian frequencies $\left(f_{i}<f_{0}\right)$ to possibly account for slow time-changing trends. Several ultradian components $\left(f_{u}>f_{o}\right)$ were also included in the model for $y_{n}$ samples. For instance, in the case of ultradian components, we added cosine terms denoted by $A_{u}\cos\left(2\pi f_{u}t_{n}+\phi_{u}\right)$, being $f_{u}$ the central frequency harmonics, symbolized by $f_{u}=uf_{0}$. The total of included infradian components in the model is denoted by

(A2) $$U_{n}=\sum_{u}A_{u}\cos\left(2\pi f_{u}t_{n}+\phi_{u}\right)$$

Given that the model proposed in this research considers ultradian components, the analysis needs to incorporate possible narrow-band fluctuations, denoted as $F_{n}^{j-}$, and $F_{n}^{j+}$ [49]. For this purpose, additional cosine terms are included, whose frequencies differ from those associated with each component in a magnitude according to the maximum spectral resolution defined by $\Delta f=f_{s}/N$, where $f_{s}$ is the sampling frequency, so that these fluctuations are determined as

(A3) $$F_{n}^{j-}=A_{j}^{-}cos\left(2\pi \left(f_{j}-\Delta f\right)t_{n}+\phi_{j}^{-}\right)$$

(A4) $$F_{n}^{j+}=A_{j}^{+}cos\left(2\pi \left(f_{j}+\Delta f\right)t_{n}+\phi_{j}^{+}\right)$$

where $j=1,2,\ldots u{}_{max}$, $f_{j}$ is the pivotal component with k=1, $f_{k}$ is the ultradian component, and $u{}_{max}$ is the maximum number of harmonics considered in the model.

For infradian components, we also incorporate cosine terms denoted by $A_{i}\cos\left(2\pi f_{i}t_{n}+\phi_{i}\right)$, where $f_{i}=i\Delta f$, and $i=1,2,\ldots i{}_{max}$. To distinguish between moderate $f_{o}$ fluctuations (low-frequency trends) and the infradian rhythms, we limited the last component to the highest integer multiple of the spectral resolution that is less than or equal to $f_{o}-2\Delta f$, therefore $i{}_{max}=\left[f_{o}/\Delta f\right]-2$, and the complete set of possible infradian components are described by

(A5) $$I_{n}=\sum_{i}A_{i}\cos\left(2\pi f_{i}t_{n}+\phi_{i}\right)$$

Finally, based on all these considerations, the complete expression of the used rhythmic model is as follows:

(A6) $$y_{n}=M+A_{0}\cos\left(2\pi f_{0}t_{n}+\phi_{0}\right)+U_{n}+I_{n}+\sum_{f_{j}}\left(F_{n}^{j-}+F_{n}^{j+}\right)+e_{n}$$

The non-significant spectral components are eliminated to reduce the uncertainty of the model, which is possible from the consecutive comparison of pairs of models, symbolized as $X{}_{A-1}$ and $X{}_{A}$. Regression model $X{}_{A}$ is obtained by adding the cosine component of Equation (A6) with its highest mean power with some criterion (for instance, in the Fourier Transform of the time series) to model $X{}_{A-1}$. A paired bootstrap hypothesis test is applied for the comparison, from the calculation of the mean square errors (MSE) of both models, represented as $e{}_{A-1}$, and $e{}_{A}$. The interested reader can consult Goya-Esteban et al. [66] for details and references therein. In brief, the relevance of the addition of each cosine component is determined by resampling with replacement of the differences between the errors, calculated by

(A7) $$\Delta E^{*}\left(b\right)=E_{A-1}^{*}\left(b\right)-E_{A}^{*}\left(b\right)$$

for each resampling, with b=1,2,3,…,B. In our experiments, we used B=2500. The asterisk symbol ${(}^{*})$ is commonly used in bootstrap literature to represent the quantities obtained from or referred to the resampling process [67]. In the hypothesis test, we considered that the addition of cosine components to the model $X{}_{A}$ was relevant if at least 95% of the $\Delta E^{*}$ values were located on the right side of zero in the estimated probability density function. Therefore, the generation of components stopped if the hypothesis test confirmed that model $X{}_{A}$ was not statistically and significantly better than model $X{}_{A-1}$.

*Scatters and Bland–Altman Plots.* If we have a time-sampling represented by a delta train,

(A8) $$m\left(t\right)=\sum_{r=1}^{M}\delta \left(t-tr\right)$$

and two signals $y_{n}\left(t\right)$ and ${\hat{y}}_{n}\left(t\right)$, then

(A9) $$y_{t_{i}}\left(t\right)=m\left(t\right)y_{n}\left(t\right)=\sum_{r=1}^{M}y_{n}\left(tr\right)\delta \left(t-tr\right)$$

(A10) $${\hat{y}}_{t_{i}}\left(t\right)=m\left(t\right){\hat{y}}_{n}\left(t\right)=\sum_{r=1}^{M}{\hat{y}}_{n}\left(tr\right)\delta \left(t-tr\right)$$

and therefore the scatterplot is the representation of the pairs

(A11) $$S\left(y_{tr},{\hat{y}}_{tr}\right)\equiv \left\{\left(y_{n}\left(tr\right),{\hat{y}}_{n}\left(tr\right)\right),\;r=1,\cdots ,R\right\}$$

*MI Between Two Variables.* Whereas MI can be defined for continuous and discrete random variables, we work here with the discrete version, assuming that we can define discrete states by quantifying the range of the environmental variables under our analysis. In this case, if $Y_{1}$ and $Y_{2}$ denote two random variables corresponding to two measured environment signals $y_{n}^{1}\left(t\right)$ and $y_{n}^{2}\left(t\right)$, which can be divided into *N* and *M* states, denoted as $y_{n}^{1}\left(i\right)$ and $y_{n}^{2}\left(j\right)$, respectively, then the MI of these random variables is given by

(A12) $$MI\left(Y_{1},Y_{2}\right)=\sum_{i}^{N}\sum_{j}^{M}p\left(y_{n}^{1}\left(i\right),y_{n}^{2}\left(j\right)\right)log\frac{p\left(y_{n}^{1}\left(i\right),y_{n}^{2}\left(j\right)\right)}{p\left(y_{n}^{1}\left(i\right)\right)p\left(y_{n}^{2}\left(j\right)\right)}$$

where $Y_{1}=\left\{y_{n}^{1}\left(i\right),i=1,2,\ldots ,N\right\}$ and $Y_{2}=\left\{y_{n}^{2}\left(j\right),j=1,2,\ldots ,M\right\}$ are the said states of the observed time signals. It can be seen that this expression is symmetric in $Y_{1}$ and $Y_{2}$ and always positive, and is equal to zero if and only if $Y_{1}$ and $Y_{2}$ are independent [68]. Its units are Shannons, although they are more commonly known as bits. Note that, if $Y_{1}$ and $Y_{2}$ are independent, then the knowledge about $Y_{1}$ does not provide any information about $Y_{2}$, and hence $MI(Y_{1},Y_{2})=0$. On the other hand, if $Y_{1}$ is a deterministic function of $Y_{2}$, and $Y_{2}$ is a deterministic function of $Y_{1}$ then all the information conveyed by $Y_{1}$ is provided by $Y_{2}$, and vice versa, in this case, the MI will equal to the entropy of $Y_{1}$, hence equal to the entropy of $Y_{2}$.

## Appendix B. Detailed Rhythmometric Model Adjustment for CO_2_ Measurements

For the description of the adjustment and validation procedure of the environmental signals modeling, we present next the CO${}_{2}$ variable as an illustrative example, because it is a noisy signal in which the adjustment is a complex procedure. Specifically, we selected the acquired samples for Node 4 of the DigiMesh network, and in this case the rhythmometry analysis gave 18 significant spectral components, 1 corresponding to the infradian rhythms $\left(f_{i}\right)$, and 17 to the ultradian ones $\left(f_{u}\right)$. The number of significant components included in the model was strongly affected by the Reg quality factor.

In the first test, the original signal was not filtered (Ord = 0), and the regularization factor was null (Reg = 0). With these considerations, the rhythmometric analysis assigned one significant infradian frequency $\left(f_{i-sig}\right)$ and only two significant ultradian frequencies $\left(f_{u-sig}\right)$. Figure A1 shows the results of the rhythmometric analysis, and the model was clearly deficient, since the adjusted signal $\hat{y_{n}}$ was often not located about the middle of the observed samples. This mismatch was mostly evident during the final section of the signal, where it was relatively constant and without trends. In the time evolution of the residuals, we observed a biased systematic error with positive values, most of them distant from zero. The Bland–Altman diagram showed here a very strong residual structure, and the *X*-axis points that represent the acquired measurements with respect to *Y*-axis that correspond to the residuals often deviated from the coincidence limits. The systematic errors represented in the histogram confirmed the loose adjustment, since the distribution was far from Gaussian and strongly asymmetrical, and even the residual range was large in comparison with the signal fluctuation range.

Figure A2 shows the relevant results from the second experiment, where the filter stage was still omitted, and we increased Reg to 10, and later to 1000. In both cases, the results were very similar. The number of significant components increased to 15 and the model adjustment was slightly improved, especially in the final sections of $\hat{y_{n}}$, where in the first test was observed a loose adjustment. In addition, the residuals on the histogram were distributed in a slightly shorter interval, and the relative frequency increased considerably for errors close to zero. The Bland–Altman structures were similar to the ones shown in the previous scenario, since the limited concordance between the measured and estimated samples persisted. To improve this modeling, Reg was increased to a much higher value (Reg = 10,000), and although the number of significant components increased to 16, the results obtained were very similar to those shown in Figure A1, as the model quality again decreased. Based on these results, we concluded that the increment of Reg by itself not necessarily improved the adjustment in such a noisy conditions, and that an efficient modeling requires also to include a mean filter, most notably in extremely noisy signals.

Finally, we applied a digital zero-phase filter to the original signal, whose maximum order was dependent on the sampling rate, noting that, for the DigiMesh and ZigBee networks, one sample was acquired approximately every 3 s, whereas, in the case of the WiFi network, one sample was acquired every 8 s. Moreover, the filter order was selected to efficiently remove undesired components without distorting the measured data trend over time. In this experiment we analyzed two cases of filtering (Ord=30 and Ord=100) to compare the results and select the most appropriate option. For both scenarios, we modified Reg, aiming to ensure that $\hat{y_{n}}$ was more persistently close to the middle of the acquired samples. After several tries, this parameter was found to be working well with values between 5 and 35. Figure A3 depicts the rhythmometric analysis results for three Reg values, one of which is outside the suggested range (Reg = 40). In this case, the signal model lost the trend with respect to the original samples in several time sections, because few significant components were assigned, the residual range of the histogram was much greater, and the negative asymmetry was mostly noticeable for the filter with Ord = 100. The adjustment was improved for the other two values (Reg = 15 and Reg = 33), and the graphic representations in both cases were similar. The residual time evolution improved considerably compared to the previous results and it was symmetrical except from the midday of Tuesday, where the CO${}_{2}$ concentration greatly increased but the model was not adjusted to follow this raise even under optimal conditions of Reg and Ord, and also the asymmetry of statistical error distribution was pronounced herein. Nevertheless, the best option was considered to filter with Ord = 100, as the relative frequencies of the histogram increased for errors closer to zero.

With this background, we see that, for variables with similar behavior, a longer period of data acquisition is required for an adequate signal processing, and then the long-term modeling can be more precise, especially if the measured signal presents spikes or decreases with considerable amplitudes. For all trial scenarios, the Bland–Altman graphs shown in Figure A4 were very similar; the concordance limits were substantially reduced in comparison with the first experiment (Reg and Ord quality parameters null); and the dispersion was symmetric and very near the limits, except in the large amplitude section, in which some few points were scattered with positive bias, due to the abrupt CO${}_{2}$ increase described before. Overall, the filter inclusion was shown to remarkably improve the modeled signal accuracy with respect to the measured data, despite the Reg factor not always was the most suitable. Based on the Bland–Altman diagrams, we confirmed that the most accurate modeling was obtained with Reg∈(15,33) and Ord = 100 because most of the points were scattered inside the concordance limits, the symmetry improved even in cases of major CO${}_{2}$ concentration, and the most distant points of the confidence band were minimal compared to the total scatter.
